# Microbial Biocontrol Agents and Natural Products Act as Salt Stress Mitigators in *Lactuca sativa* L.

**DOI:** 10.3390/plants13172505

**Published:** 2024-09-06

**Authors:** Claudio Caprari, Antonio Bucci, Anastasia C. Ciotola, Carmine Del Grosso, Ida Dell’Edera, Sabrina Di Bartolomeo, Danilo Di Pilla, Fabio Divino, Paola Fortini, Pamela Monaco, Davide Palmieri, Michele Petraroia, Luca Quaranta, Giuseppe Lima, Giancarlo Ranalli

**Affiliations:** 1 Department of Biosciences and Territory, University of Molise, C.da Fonte Lappone snc, 86090 Pesche, Italy; antonio.bucci@unimol.it (A.B.); a.ciotola@studenti.unimol.it (A.C.C.); i.delledera@studenti.unimol.it (I.D.); sabrina.dibartolomeo@unimol.it (S.D.B.); d.dipilla1@studenti.unimol.it (D.D.P.); fabio.divino@unimol.it (F.D.); fortini@unimol.it (P.F.); pamela.monaco@unimol.it (P.M.); m.petraroia2@studenti.unimol.it (M.P.); l.quaranta1@studenti.unimol.it (L.Q.); ranalli@unimol.it (G.R.); 2 Department of Agriculture, Environment and Food Sciences, University of Molise, Via F. De Sanctis snc, 86100 Campobasso, Italy; c.delgrosso2@studenti.unimol.it (C.D.G.); davide.palmieri@unimol.it (D.P.); lima@unimol.it (G.L.); 3 Institute for Sustainable Plant Protection, National Research Council (CNR), 70126 Bari, Italy; 4 Department of Mathematics and Statistics, University of Jyväskylä, 40014 Jyväskylä, Finland

**Keywords:** abiotic stress, biocontrol, stress mitigation, antagonistic microorganism, plant biostimulation, *Lactuca sativa* var. *romana*

## Abstract

One of the major problems related to climate change is the increase in land area affected by higher salt concentrations and desertification. Finding economically and environmentally friendly sustainable solutions that effectively mitigate salt stress damage to plants is of great importance. In our work, some natural products and microbial biocontrol agents were evaluated for their long-term effectiveness in reducing salt stress in lettuce (*Lactuca sativa* L. var. *romana*) plants. Fourteen different treatments applied to soil pots, with and without salt stress, were analyzed using biometric (leaf and root length and width), physiological (chlorophyll and proline content), and morphological (microscopic preparations) techniques and NGS to study the microbial communities in the soil of plants subjected to different treatments. Under our long-term experimental conditions (90 days), the results showed that salt stress negatively affected plant growth. The statistical analysis showed a high variability in the responses of the different biostimulant treatments. Notably, the biocontrol agents *Papiliotrema terrestris* (strain PT22AV), *Bacillus amyloliquefaciens* (strain B07), and *Rahnella aquatilis* (strain 36) can act as salt stress mitigators in *L. sativa*. These findings suggest that both microbial biocontrol agents and certain natural products hold promise for reducing the adverse effects of salt stress on plants.

## 1. Introduction

Lettuce (*Lactuca sativa* L. var. *romana*), a member of the Asteraceae family, is one of the most popular vegetables. It is becoming increasingly accepted by consumers because it is healthy and easy to prepare, especially in salads. The most used parts are the leaves, followed by the stems (for juice) and seeds (for seed mixes) [1]. The popularity achieved by lettuce, which is consumed throughout the world, is also because it thrives best at temperatures between 7 and 24 °C. In addition, since lettuce is a long-day plant, varieties that are not affected by the length of the day have been selected to allow rich foliage to grow in all light conditions [1,2,3]. Lettuce leaves are a healthy source of glycosylated flavonoids, phenolic acids, carotenoids, vitamin B groups, ascorbic acid, tocopherols, sesquiterpene lactones, minerals, and fiber; they are essentially a good source of vitamins and antioxidants. These phytochemicals are very useful and beneficial to the human body. They also have a positive effect on controlling blood cholesterol levels [4,5,6,7]. Lettuce plants are moderately sensitive to salinity stress, which negatively affects their productivity [8].

Climate change is becoming a critical factor, since it has a significant impact on all areas of human activity, with negative effects on the environment in many parts of the world, on the life and health of people, and on various sectors of the economy [9]. Agriculture is particularly vulnerable to the impact of climate change, as it is one of the most weather-dependent sectors of the economy. The negative effects of climate change, coupled with the rapid melting of glaciers and the prolonged periods of drought, have led to a significant decline in agricultural development worldwide, especially in semi-arid regions [10,11].

Among plant abiotic stresses, salinity is particularly harmful to lettuce plants, since these plants are moderately sensitive to salinity stress; high salinity negatively affects lettuce productivity. Salinity causes significant damage to biodiversity, ecosystems, human health, and natural resources, posing a serious threat to modern agriculture [12,13]. Salinity stress affects plants in several ways: (1) it decreases the water potential at the rhizosphere level, resulting in a plant water deficit; (2) it induces phytotoxicity due to the accumulation of ions such as Na^+^ and Cl^−^, usually leading to the abundant production of reactive oxygen species (ROS) with the impairment of physiological processes, especially photosynthesis and protein synthesis [14,15]; and (3) it decreases plant nutrient uptake and transport [16]. However, the primary factor contributing to salt accumulation in agricultural soils is the high concentration of salts in irrigation water [17]. Finally, increased soil salinity can also result from plant transpiration or evaporation from the soil surface, leading to higher water evapotranspiration and further soil salinization [9]. To make effective decisions for sustainable agricultural management, it is essential to consider these climate changes and assess the impact of air temperature on soil salinity [18].

Over the last 5 years (2020–2024), a constantly increasing number of papers on biostimulants and plant growth have been published (from 193 in 2020 to 277 in 2024, according to Scopus 6/2024), which confirms the interest in this matter. Plant growth-promoting microorganisms (PGPMs) are a potential source of both alternative agrochemicals (fertilizers and pesticides) and environmental stress mitigators [19,20,21]. The same authors adopted lettuce as a plant model to describe the biocontrol activity of *Azotobacter chroococcum* against fungal infection. They hypothesized a double-mode action for this organism as both an antagonist agent and a biostimulant [19]. Moreover, an interesting research field is the biological control of plant growth, including the suppression of pathogens by beneficial microorganisms that have been isolated and adopted in several studies, both in vitro and in vivo. In recent years (2020–2024), a constant number of papers that have used “biological control” and “plant growth” as keywords have been published (1591 in 2020; 1719 in 2021; 1706 in 2022; 1730 in 2023; and 900 in 2024, according to Scopus 6/2024) [22].

Microbial biocontrol agents (BCAs) represent a promising alternative to chemical control agents, with several BCA formulations already available for practical use [23,24]. Recently, newly characterized lipopeptides from *Bacillus amyloliquefaciens* exhibited strong inhibitory activity against *Fusarium oxysporum*, indicating a potential role in biocontrol activity [25]. In a recent study, the biocontrol agent *Papiliotrema terrestris*, strain PT22AV, was developed as the active component of a granular formulation already tested as a biological fungicide in several trials [26]. Moreover, the endophytic rhizobacterium *Rahnella aquatilis*, strain 36, controlled the root-infecting fungal pathogen *F. oxysporum* [27].

This work represents a cohort (also called “follow-up”) study based on 14 different treatments carried out over a long period of time (up to 90 days) on *L. sativa* var. *romana* plants with and without NaCl salinity stress. Specifically, the overall objectives of our work were to compare several natural products and to verify whether three previously characterized microbial biocontrol agents (*P. terrestris*, strain PT22AV; *B. amyloliquefaciens*, strain B07; and *R. aquatilis*, strain 36) could act as salt stress-mitigating agents on *L. sativa*. This study involved the following steps: (i) verifying the effects of different microbial biocontrol agents, natural products, and biostimulant applications on lettuce growth and mortality; (ii) determining the attenuation by these applications of induced salt stress by measuring physiological performances; and (iii) analyzing the microbial community in the lettuce’s peaty substrate, both treated and untreated with salt.

## 2. Results

### 2.1. Mortality Analysis

**Figure 1:**
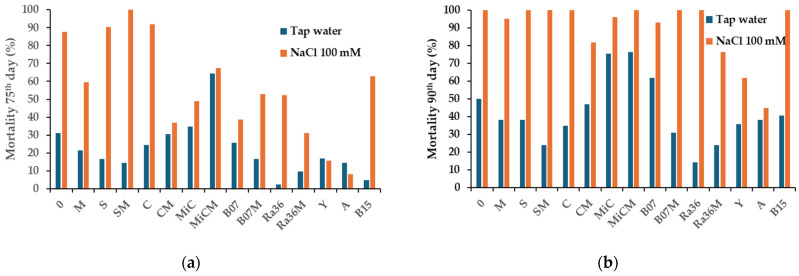
Mortality cumulative incidence of lettuce plants on (**a**) 75th and (**b**) 90th days after exposure to saline stress (controls vs. treatments). Legend: 0 = control without treatments; M = molasses; S: soil; SM = S + M; C = compost; CM = C + M; MiC = micro compost mix; MiCM = MiC + M; B07 = *B. amyloliquefaciens* strain B07; B07M = B07 + M; Ra36 = *R. aquatilis* strain 36; Ra36M = Ra36 + M; Y = *P. terrestris* strain PT22AV; A = Activeg; B15 = Bioalga 15.

Plant mortality was evaluated in terms of cumulative incidence, i.e., the number of plants that died within a given time divided by the total number of plants at the beginning of the study, expressed as a percentage. Specifically, the 75th and 90th days were considered significant time points. Figure 1 summarizes the mortality data of the plants on the 75th and 90th days subjected to different treatments with protein hydrolysates and cultures of microorganisms (bacteria and yeasts). The 75th day was considered because it was the time of 100% mortality for the first plot of plants (SM treatment), whereas the 90th day was the last monitoring time of the experiment.

**Figure 2:**
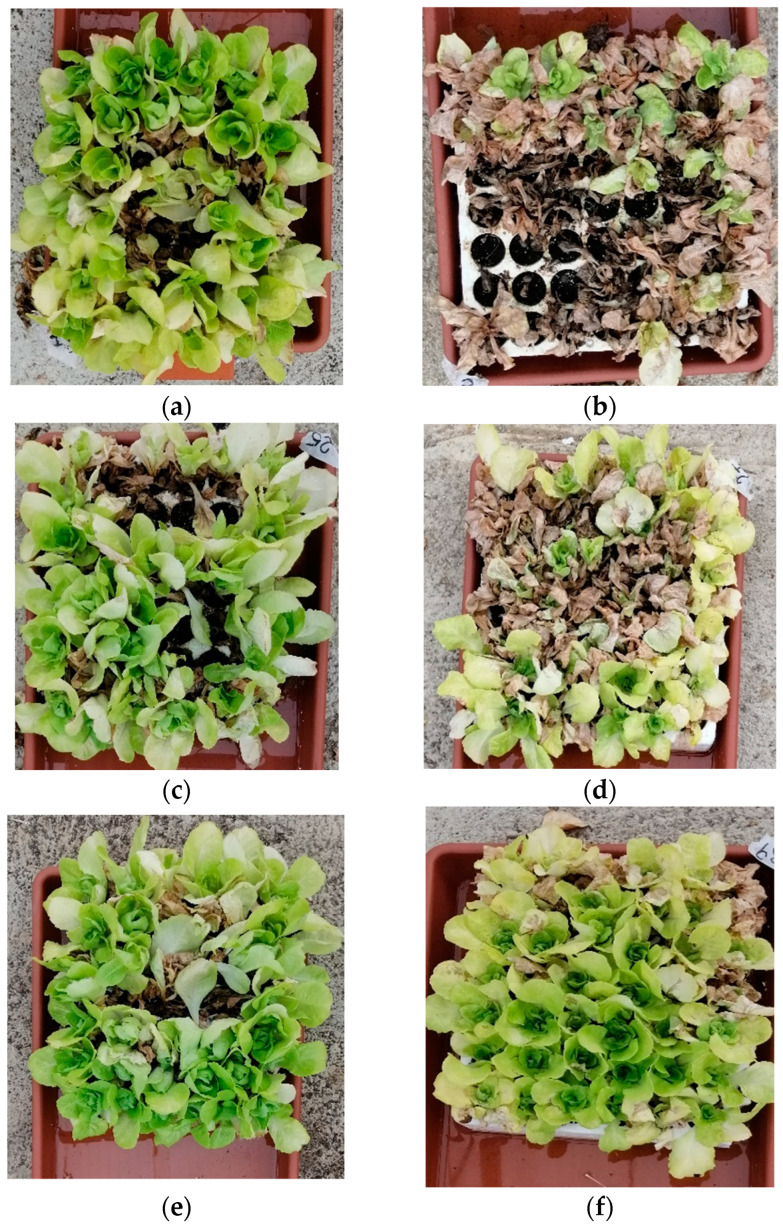
Mortality of lettuce plants on 75th day: (**a**) tap water as control, (**b**) 100 mM NaCl solution as control, (**c**) B07 treatment with tap water, (**d**) B07 treatment with 100 mM NaCl, (**e**) *P. terrestris* strain PT22AV treatment with tap water, and (**f**) *P. terrestris* strain PT22AV treatment with 100 mM NaCl.

As an example, Figure 2c–f show the results of the treatments of *L. sativa* with the microbial strain B07 and *P. terrestris* PT22AV. These two treatments showed a marked mitigating effect against salt stress on day 75. The survival was still high compared to the control plants (Figure 2a,b).

**Figure 3:**
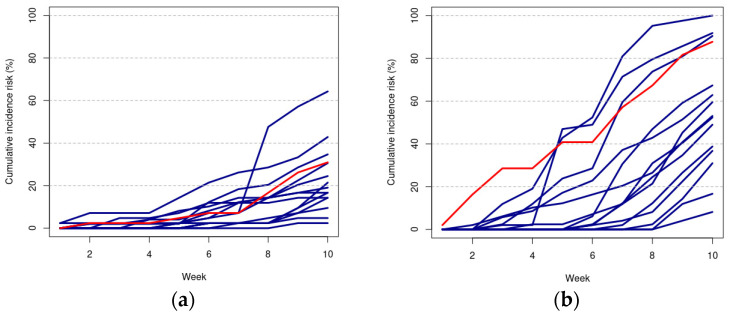
In blue, the CIR trajectories for each treatment are shown, while in red, the CIR trajectories of the respective control treatment are shown, across the ten weeks, under the experimental conditions with only tap water (**a**) and with the addition of NaCl (**b**).

In Figure 3, the blue lines represent the cumulative incidence risk (CIR, %) trajectories for each treatment, which were reported across a time span of ten weeks under the experimental conditions with only tap water (panel a) and with the addition of NaCl (panel b). In both panels of Figure 3, the CIRs calculated with respect to the respective control treatment (denoted by j = 0) are reported in the color red. Note that the higher the cumulative incidence risk of mortality, the lower the level of protection of the respective treatment.

Then, we considered the Z test to control for the potential differences between the CIRs of each treatment and the respective control CIRs across the ten weeks. In other words, with respect to the plots in Figure 3, we controlled how significantly higher or lower the levels of the blue lines were each week compared to the levels of the respective red line.

**Figure 4:**
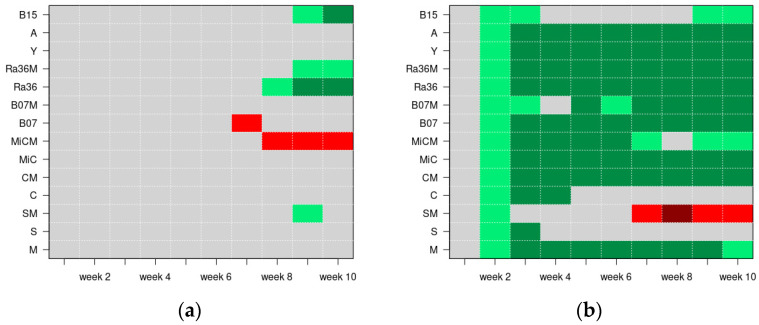
The levels of significance of the Z tests, across the ten weeks, under the experimental conditions with only tap water (**a**) and with the addition of NaCl (**b**): the pixels in dark green and dark red show the CIRs, respectively, lower or higher than the control CIRs (significance 0.001); the pixels in light green and light red show the CIRs, respectively, lower or higher than the control CIRs (significance 0.01); and the pixels in gray show no significant difference between the treatment and control CIRs. Legend: M = molasses; S: soil; SM = S + M; C = compost; CM = C + M; MiC = micro compost mix; MiCM = MiC + M; B07 = *B. amyloliquefaciens* strain B07; B07M = B07 + M; Ra36 = *R. aquatilis* strain 36; Ra36M = Ra36 + M; Y = *P. terrestris* strain PT22AV; A = Activeg; B15 = Bioalga 15.

In Figure 4, panel (a) shows the data for only tap water and panel (b) shows the data for the addition of NaCl. The levels of significance of the Z tests are reported as colored images for each treatment and each week: the pixels are in dark green and dark red when the CIR_jt_ was, respectively, lower or higher than the CIR_0t_ with a significance of 0.001; the pixels are in light green and light red when the CIR_jt_ was, respectively, lower or higher than the CIR_0t_ with a significance of 0.01; and finally, the pixels are in gray when there was no significant difference between the CIR_jt_ and CIR_0t_.

Further, to better assess the effectiveness of each treatment, the respective CIRs (in percentages) of the treatments and controls are reported in Table 1 for the 10th week under the experimental conditions with only tap water and with the addition of NaCl.

From this Table 1, it can be seen that only a few treatments were effective at mitigating NaCl-induced stress. Among the best were the two treatments with the biostimulant Activeg (CIR = 8.2%) and the treatment with the biocontrol agent Y (*P. terrestris*). The latter had a CIR of 16.7%. The other treatments, such as B07 (*B. amyloliquefaciens*) and RA36M (*R. aquatilis* strain 36 + M), were less effective. Molasses (M) added alone to the compost treatments induced a CIR reduction.

The experimental design (Section 4.1) included fourteen different treatments. The treatments were applied to both the control and salt-stressed lettuce plants. We focused on the three biocontrol agents and Activeg for the subsequent in vivo analysis because not all treatments proved effective. We also reported on the molasses treatment because it was an additional part of the biocontrol agent treatments.

### 2.2. Biometric Measures

The biometric data (see Section 4.3.2) of the whole lettuce plants were obtained in a non-destructive analysis by measuring the leaf size, height, and width on the 60th day, as shown in Table 2.

Student’s *t*-test showed significant effects of the salinity level and biostimulant applications, but not for all of the growth parameters evaluated. The B07M, Ra36, and Ra36M treatments showed a significant reduction in the height of the lettuce leaves, while only the Activeg and Y treatments induced a significant increase in the height when comparing the control treatment with the salt treatment. Non-significant variations were recorded in most of the tests regarding the width of the lettuce leaves. The Ra36M and Activeg treatments were the exceptions.

### 2.3. Chlorophyll Content Analysis

**Figure 5:**
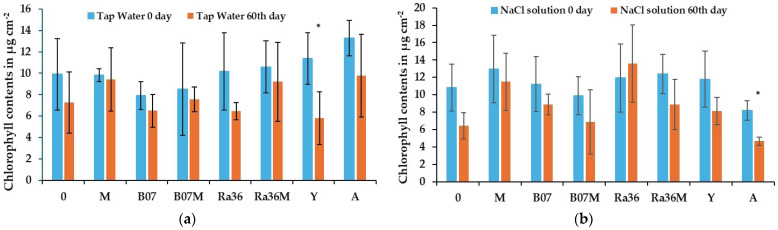
Chlorophyll contents in µg cm^−2^, mean ± SD (n = 3), of treated plants and their response to salt stress and control treatments. Tap water (**a**) and 100 mM NaCl (**b**), at two times (0 and 60th day). Significant difference of 0 day-treated plants with respect to 60th day-treated plants, based on Student’s *t*-test (* *p* ≤ 0.05). Legend: 0 = control without treatments; M = molasses; B07 = *B. amyloliquefaciens* strain B07; B07M = B07 + M; Ra36 = *R. aquatilis* strain 36; Ra36M = Ra36 + M; Y = *P. terrestris* strain PT22AV; A = Activeg.

The relative chlorophyll content in the leaves of the water-only controls and of the NaCl-treated plants on the 60th day of exposure is shown in Figure 5a,b. The chlorophyll content decreased with time under both the stress and non-stress conditions. Decreases in the chlorophyll content of up to several percentage points occurred under both conditions, but only the *P. terrestris* treatment (Figure 5a) was found to be statistically significant in the water-only samples. For the salt-stressed samples, only the treatment with Activeg was statistically significant (Figure 5b). The Ra36 sample showed an increase in the chlorophyll content in the salt-stressed samples, although the difference was not significant, contrary to the general trend. Finally, regarding the decrease in the chlorophyll content caused by the action of salt stress, the best performance was observed for the treatment with Ra36. The sample treated with *P. terrestris* showed a good effect in buffering the decrease in the chlorophyll content.

### 2.4. Proline Assay

A proline assay was performed on lettuce leaves only at the end of the long-term study (on day 90), according to the initial experimental design, which included non-destructive parameters and analyses. Under our conditions, in many of the lettuce leaves subjected to the treatments, the proline assays showed values close to or below the limit of the sensitivity of the calibration curve (<0.17 µg/mL). However, the proline assay on the lettuce leaves associated with the Ra36M, Y, and A biotreatments showed slightly higher values ranging from 0.90 to 4.5 µg/mL, but no statistical differences were recorded. These results suggest that to obtain useful information on the true effects of salinity stress and the real efficacy of the compared biostimulants, performing a proline assay in lettuce leaves after the late stage of plant growth (90 days) is not suitable.

### 2.5. Confocal Microscope Observations

**Figure 6:**
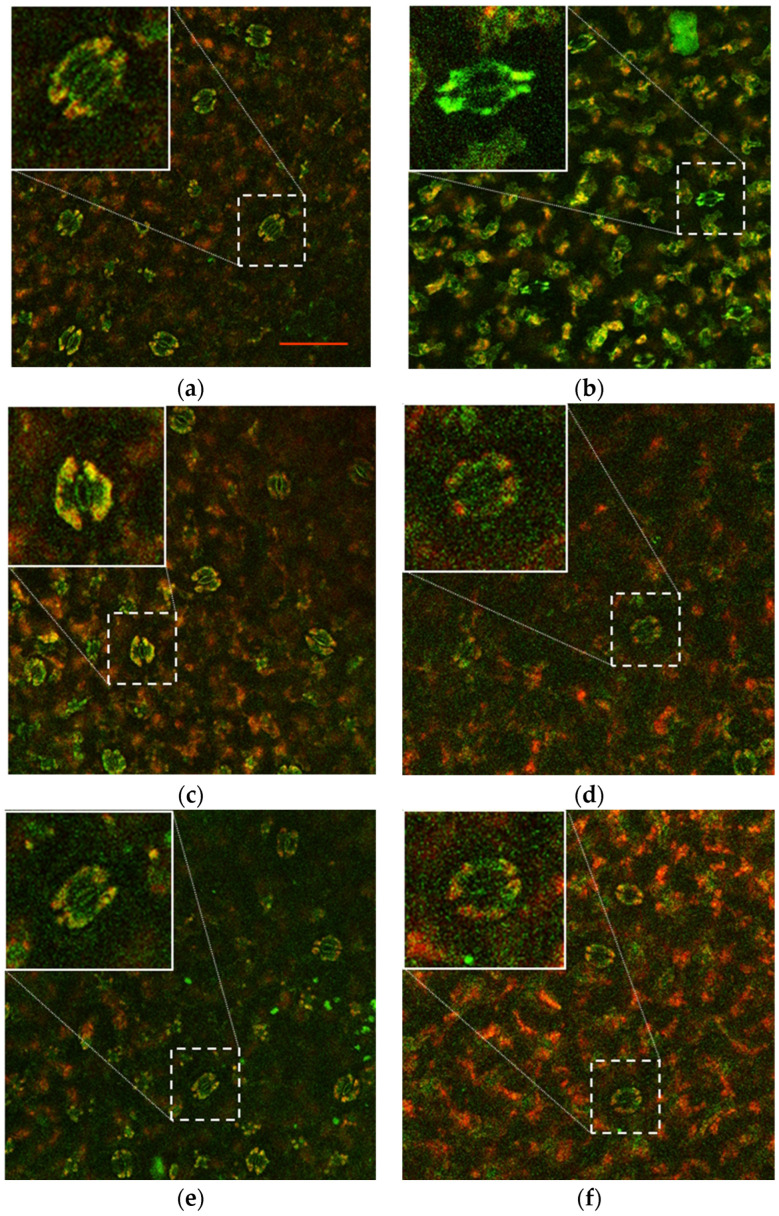
Confocal microscope observations of lettuce leaves: (**a**) tap water control, (**b**) 100 mM NaCl solution control, (**c**) B07 treatment with tap water, (**d**) B07 treatment with 100 mM NaCl, (**e**) *P. terrestris* strain PT22AV treatment with tap water, and (**f**) *P. terrestris* strain PT22AV treatment with 100 mM NaCl. Red and green fluorescence signals were captured after 400–600 nm laser excitation of plants pigments. Representative images of at least 10 fields are shown. Scale bar: 100 µm. Inset shows 3× magnification of stomata.

The autofluorescence of lettuce leaf samples was also observed using a confocal microscope to evaluate the modulation of the tissue structure in response to the different conditions. As shown in Figure 6b, the leaves of NaCl-treated plants presented an abnormal stomatal structure and a different fluorescence pattern compared to the control plants on the 75th day (Figure 6a), reflecting the response of leaves to the strong salt stress. However, these effects appeared to be strongly mitigated when the plants were grown in the presence of the microbial agents B07 (Figure 6d) and *P. terrestris* (Figure 6f) compared to their tap water controls (Figure 6c and Figure 6e, respectively).

### 2.6. Root Analysis

**Figure 7:**
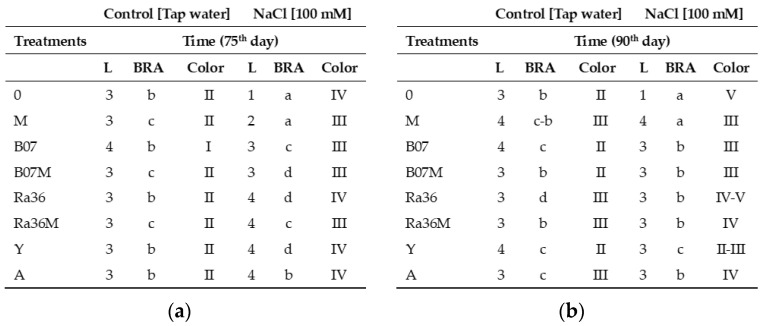
Length, branching, and root color of lettuce plants in response to salt stress (tap water and 100 mM NaCl) on 75th day (**a**) and on 90th day (**b**). L = length (1 < 0.5 cm; 2 = 0.6–1.0 cm; 3 = 1.1–2.0 cm; 4 = 2.1–3.0 cm; 5 > 3.1 cm). BRA = branching (a < 0.5 cm; b = 0.6–1.0 cm; c = 1.1–2.0 cm; d = 2.1–3.0 cm; e > 3.1 cm). Color (I, white; II, creme; III, light brown; IV, dark brown; V, black). Treatments: 0 = control without treatments; M = molasses; B07 = *B. amyloliquefaciens* strain B07; B07M = B07 + M; Ra36 = *R. aquatilis* strain 36; Ra36M = Ra36 + M; Y = *P. terrestris* strain PT22AV; A = Activeg.

Root observations on the 75th and 90th days are reported in Figure 7 and Figure S1.

The roots of the lettuce seedlings grown with H_2_O alone (control) compared to all of the treatments showed the following (Figure 7a and Figure S1a): (a) In 12 cases, a root length between 1.1 and 2.0 cm was observed and (b) only the B07 treatment revealed a root length between 2.1 and 3.0 cm. The color of the root system was generally cream except for the B07-treated roots, which appeared white (Figure 7a and Figure S1c). Furthermore, root branching in 10 treatments showed values between 0.6 and 1.0 cm. The remaining three treatments (M, B07M, and Ra36M) showed higher branching (1.1–2.0 cm). These values indicate a healthy root system. On the other hand, the lettuce roots treated with 0.1 M NaCl showed high variability in their length between treatments. A value of less than 0.5 cm was observed for the treatment with NaCl alone (Figure 7a and Figure S1b); a value between 0.6 and 1.0 cm was observed for the treatment with M. Values between 1.1 and 2.0 cm were recorded for the B07 and B07M treatments (Figure 7a and Figure S1d). Finally, the highest root length values were observed for the Ra36, Ra36M, Y, and A treatments. Similarly, higher values of root branching (2.1–3.0 cm) appeared in the Ra36, Ra36M, and Y treatments, where the roots were light or dark brown in color, unbranched, and attached to the bottom of the polystyrene tray, indicating a suboptimal state of the root system, as shown in Figure 7a,b. The results in Figure 7b show further deterioration in the parameters considered (length, branching, and color) for the lettuce roots on day 90 of the saline treatments, compared with the data from day 75.

### 2.7. Viable Microbial Count

The viable microbial count results for the pot soil are reported in Table 3.

Considering all ofmthe treatments, a significant difference was shown only for the 0 (control) and A (Activeg) treatments for the total viable bacterial count (Table 3). The control showed a lower number of microbes in the NaCl-treated sample. In the Activeg treatment, we observed a higher value of the TVBC with a low amount of the treatment. Only the 0, Ra36M, and Y (*P. terrestris*) treatments showed significant differences in the number of spore-forming bacteria. The control and Y treatments followed the same trend, with a reduction in the microbial count in the stressed samples; otherwise, an increased value was found for the stressed Ra36M treatment, and high values were obtained for both tap water and the saline solution. Finally, when *P. terrestris* was added to the NaCl treatment, the result was a significant increase in the number of yeast and fungal cells, and the final microbe number was close to 10^7^ CFU g^−1^ fw^−1^.

### 2.8. Microbial Community Analysis

Soil bacterial communities of lettuce seedlings subjected to salt stress (samples: 0_NaCl_2 and 0_NaCl_3) and of stressed plants treated with the B07 strain (samples: B07_NaCl_1, B07_NaCl_2, and B07_NaCl_3) were examined using an NGS (next-generation sequencing) analysis of 16S rRNA gene amplicons (V3-V4 regions). The corresponding control samples (0_H_2_O_1, 0_H_2_O_3 and B07_H_2_O_2, B07_H_2_O_3, respectively) were also analyzed. The total number of final reads for each of these samples is reported in Table S2.

**Figure 8:**
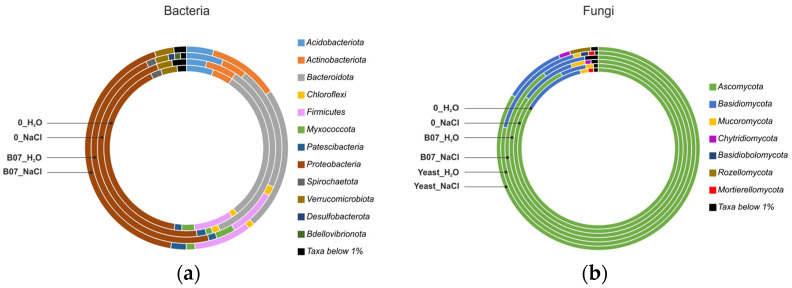
Bacterial (**a**) and fungal (**b**) community composition in controls and treatments at phylum level.

To examine the taxonomic composition of the lettuce soil bacterial communities according to the treatment type, the relative abundances of the different taxa were averaged for each experimental group. Overall, twelve bacterial phyla showed relative abundance values higher than 1%. With percentages between 40.45 and 46.81% and between 21.14 and 34.61%, respectively, *Proteobacteria* and *Bacteroidota* were among the most represented phyla, followed by *Firmicutes*, *Acidobacteriota*, and *Actinobacteriota* (Figure 8a).

An important reduction in the relative abundance of *Bacteroidota* was observed in the soil microbial communities of lettuce seedlings treated with the B07 strain (B07_H_2_O and B07_NaCl groups) compared to the 0_H_2_O- and 0_NaCl-associated bacterial communities. Indeed, the relative abundance of this phylum in the 0_H_2_O and 0_NaCl samples was 30.26% and 34.61%, respectively, whereas in the samples B07_H_2_O and B07_NaCl, it decreased to 21.14% and 22.91%, respectively.

With regards to *Firmicutes* and *Actinobacteriota*, interesting differences were observed based on the experimental groups. The *Firmicutes* percentage was significantly lower in the soil samples of salt-stressed plants (0_NaCl group = 0.62%) compared to the other sample groups (0_H_2_O = 7.68%; B07_H_2_O = 8.26%; B07_NaCl = 9.14%).

The *Actinobacteriota* phylum—which showed a relative abundance between 4.00 and 5.59% in the 0_H_2_O, 0_NaCl, and B07_H_2_O samples—reached 11.23% in the soil of stressed *L. sativa* L. seedlings treated with the B07 strain (B07_NaCl).

*Acidobacteriota* exhibited higher values of relative abundance in the samples not subjected to salt stress, with a difference (Δ) of 1.61% between the 0_H_2_O and 0_NaCl groups and 1.96% between the B07_H_2_O and B07_NaCl groups.

**Figure 9:**
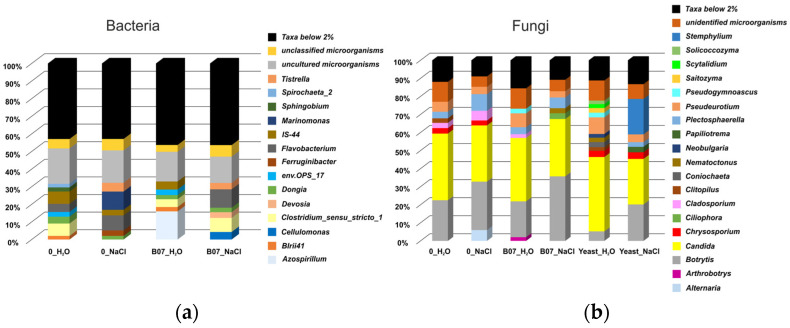
Bacterial (**a**) and fungal (**b**) community composition in controls and treatments at genus level.

An analysis of the microbial community composition at the genus taxonomic level revealed that uncultured and unclassified bacteria ranked first in all of the examined groups of lettuce soil (Figure 9a). More specifically, the average relative abundance of uncultured genera ranged between 14.94 and 20.10%, with a progressive reduction moving from samples 0_H_2_O (20.10%) to 0_NaCl (18.41%), B07_H_2_O (16.85%), and B07_NaCl (14.94%). The bacteria not classified at the genus level varied between 3.88% (B07_H_2_O) and 6.55% (B07_NaCl).

*Flavobacterium*, *Dongia*, *Devosia*, and *Bauldia* were among the most represented genera in all of the analyzed groups. The relative abundance of *Flavobacterium* in the salt-stressed samples was higher than in the non-stressed ones, reaching percentages of 8.55% and 10.47% in the NaCl- and B07_NaCl-associated microbial communities, compared to 4.60% for 0_H_2_O and 1.39% for B07_H_2_O. For both *Devosia* and *Bauldia*, greater relative abundance values were observed in the B07_NaCl group (3.27% and 1.99%, respectively). The 0_H_2_O control group showed the highest percentage of bacteria belonging to the *Dongia* (3.91%) and IS-44 (7.01%) genera, as well as a relatively high abundance of *Clostridium* species (≈7.01%). Regarding the latter genus, it is interesting to note that although it was very poorly represented in the 0_NaCl samples (average relative abundance = 0.02%), it reached a percentage of ≈7.98% in the soil bacterial communities of salt-stressed seedlings treated with the B07 strain.

On the contrary, the *Marinomonas* genus, which was absent in the 0_H_2_O, B07_H_2_O, and B07_NaCl samples, was among the most represented genera in the 0_NaCl group. Similarly, *Azospirillum* (absent or with a relative abundance of <0.4% in the other groups) constituted about 16% of the bacterial communities of the lettuce soil samples treated with the B07 strain (Figure 9a).

In addition to the bacterial communities, the fungal communities of 18 pot soil samples collected from *L. sativa* L. seedlings subjected to the treatments summarized in Table S1 were analyzed using ITS2 region sequencing. The total number of final reads ranged between 38,252 (sample B07_H_2_O_2) and 74,014 (sample Yeast_NaCl_2; Table S3).

For both bacteria and fungi, the average relative abundance values of the different taxa were calculated for each experimental group. The NGS analysis revealed the presence of a limited number of phyla with relative abundance percentages above 1% (see Figure 8b).

*Ascomycota* was the dominant phylum in all of the examined samples, with average abundance values between 78.51% (yeast sample group) and 92.67% (NaCl sample group). Overall, fungi belonging to the *Ascomycota* phylum were more abundant in the soil mycobiota of salt-stressed plants (the 0_NaCl, B07_NaCl, and Yeast_NaCl samples) compared to the corresponding controls (0_H_2_O, B07_H_2_O, and Yeast_H_2_O, respectively).

A different trend was observed for fungi belonging to the *Basidiomycota* phylum, which showed lower relative abundance values in the soil samples of seedlings subjected to salt stress. However, *Basidiomycota* ranked second out of all of the investigated fungal communities, with percentages ranging between 4.95% (0_NaCl) and 17.35% (Yeast_H_2_O).

*Candida*, *Botrytis*, *Pseudeurotium*, *Plectosphaerella*, *Chrysosporium*, *Cladosporium*, and *Scytalidium* were among the fungal genera with a relative abundance greater than 1% in all of the examined groups (see Figure 9b). *Candida* and *Botrytis* represented an important fraction of the lettuce soil fungal communities. Indeed, fungi belonging to the *Candida* genus contributed 25.21–41.18% to the mycobiota composition, with a reduction in their relative abundance values in the salt-stressed samples (considerable in the case of yeast-treated seedlings). The genus *Botrytis* varied between 5.26 and 35.73%, showing higher percentages in the soil samples of lettuce treated with NaCl. As can be seen in Figure 9b, a part of the mycobiota (5.75–11.28%) was represented by fungi unidentified at the genus level.

Compared to the other samples, *Pseudeurotium* and *Plectosphaerella* were more copious in the Yeast_H_2_O and 0_NaCl sample groups, respectively, with relative abundance values of 9.20% and 9.23%.

Interestingly, the *Stemphylium* genus (which was overall poorly represented) reached a percentage of 19.63% in the Yeast_NaCl group. Similarly, *Alternaria*, which constituted 5.99% of the 0_NaCl sample’s mycobiota, showed an average relative abundance of <1% in the other soil samples. The rarefaction analysis, a measure used to estimate the alpha diversity in samples and gauge whether or not the sequencing efforts captured the microbial diversity, showed a higher fungal biodiversity in the soils not subjected to salt stress (average Shannon index values for the groups 0_H_2_0, B07_H_2_0, and Yeast_H_2_0 ranging from 4.76 to 5.28) compared to those treated with NaCl at a concentration of 100 mM (average Shannon index values for the groups 0_NaCl, B07_NaCl, and Yeast_NaCl ranging from 3.94 to 4.45).

**Figure 10:**
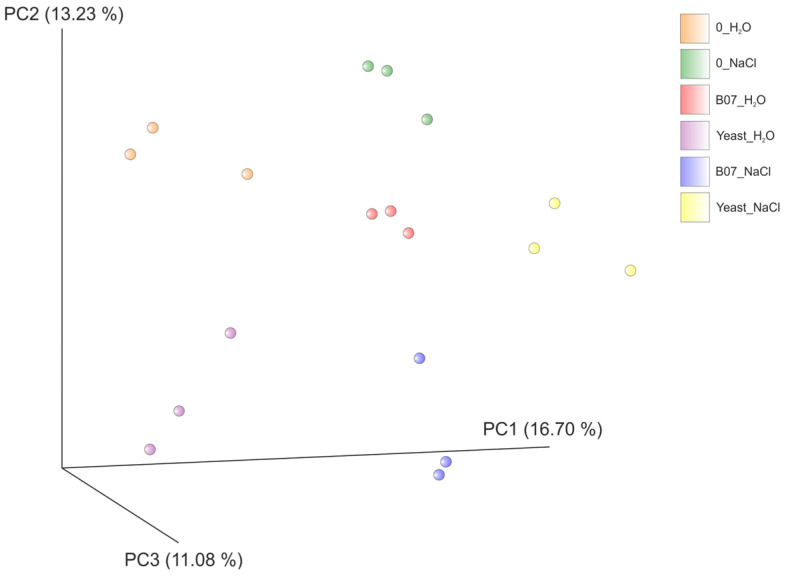
Principal coordinate analysis (PCoA) of fungal communities. The plot was generated using the Jaccard metric.

The principal coordinate analysis (PCoA) results based on the Jaccard metric highlighted the variation among the experimental groups, although intra-group variability/dispersion was also observed, as shown in Figure 10.

### 2.9. Environmental Impact, CO_2_-Equivalent Emissions, and Mitigation Actions

According to previous works, here, we report an evaluation of the environmental impact related to this study, the CO_2_-equivalent emissions, and the mitigation actions by new plantations [28,29].

The evaluation of the environmental impact of this manuscript, in terms of the social cost and CO_2_-equivalent emissions, resulted in EUR 12.0 and about 300 kg CO_2_ equivalent, respectively. This influenced us to provide a new plantation of *Carpinus* plants for mitigation actions. *C. betulus* and *C. orientalis* were used, due to them both being autochthonous species with an environmental adaptation ability. A QR code tag linked to this manuscript was added to each tree planted in Green Park—Campus Unimol (41°60′76″ N, 14°26′50″ E).

## 3. Discussion

The defined experimental design was conservative. It did not include destructive investigations such as the determination of plant biomass or the extraction of chlorophyll using chemical methods. Therefore, our experimental design favored the use of other biometric–vegetative indicators, such as (i) leaf width and length; (ii) root system length, branching, and coloration; (iii) the cumulative incidence risk (CIR), which was obtained by measuring the weekly mortality cumulative incidence of the lettuce seedlings; and (iv) the chlorophyll content, which was determined using the AtLeaf CHL PLUS tool (FT Green LLC, Wilmington, DE, USA). Confocal microscopy was also performed on a representative aliquot of lettuce leaves from each Styrofoam cassette. Following the experiments, the microbial communities of the pot soil used as a growing medium for the lettuce seedlings were analyzed. The analyses performed on epigeal (leaf) and hypogean (root and soil) fractions are discussed below.

### 3.1. Effects of Microbial Biocontrol Agents and Biostimulants on Reducing Mortality and Salinity Stress in Lettuce Leaves

The use of the cumulative incidence risk (CIR) showed a clear and positive performance for the biostimulant Activeg (Figure 3 and Figure 4). In addition, the CIR indicated that the biocontrol agents, sometimes enriched with molasses, played an additional potential role in mitigating salt stress, but at a lower intensity compared to the other biostimulants tested in this work [30,31]. Furthermore, recent studies have proposed using legume protein hydrolysates not only as biostimulants, but also as potential functional foods with minimal environmental impact [32].

Among the biometric indicators adopted, a positive performance in terms of the development of the lettuce plants was recorded on day 60 after the addition of the biostimulant Activeg (A) (see Table 2). In fact, under salt stress, the *Fabaceae* extract solution provided statistically significant increases in leaf height (increases of more than 20%), in comparison to both the control (H_2_O only) and the other treatments. In contrast, significant reductions in lettuce leaf height were observed with the use of other biostimulants (M, S, MS, and C) and biocontrol agents (B07, Ra36, Ra36M, and Y) [30,33,34,35,36].

Under the experimental conditions we used, the results showed a significant decrease in the chlorophyll content only in the samples subjected to salt stress, such as the salt control and the treatment with Activeg. The data showed a decrease in the chlorophyll content ranging from 40% to 43% (Figure 5). The results obtained here agree with the data already reported by other authors [37,38,39]. The lettuce seedlings treated with *P. terrestris* showed a decrease in their chlorophyll content of 49 percent in the control sample with the source water, while it was only 31 percent in the sample treated with the saline solution. These results agree with those of Yasar et al. [40], who stated that growth retardation, a typical symptom of salinity damage to plants, is due to inhibited cell elongation. In addition, a comparison of salt-sensitive and salt-resistant pumpkin genotypes [41] hypothesized the role of chlorophyllase activity and oxidative membrane damage induced by salt stress.

The Ra36 treatment resulted in a non-significant increase in the chlorophyll content compared to the H_2_O control. These data are consistent with the increase in leaf biometric measurements. The four treatments with molasses as the biostimulant (M, CM, SM, and MiCM) also showed no significant change. The data on the chlorophyll content in the molasses (M) treatments did not result in reduced plant mortality on the 75th day compared to the treatments with biocontrol agents such as Ra36, B07, and Y. The exception was the treatment with Activeg, which has already been reported in the literature [42] and which resulted in the best survival of the lettuce plants on both the 75th and 90th days of the saline treatment (Figure 1 and Figure 2). Furthermore, the biostimulant Activeg led to a reduction in transplant stress in the field [43,44,45]. Therefore, the biometric data on leaf height reported in Table 2 confirm the positive effect of the mitigating and biostimulating treatments [43,46]. Under our experimental conditions, the total chlorophyll content did not appear to be a useful marker for assessing the long-term response of salt-stressed lettuce plants. Most of the changes in the concentration did not appear to be significantly correlated with the CIR.

Salt stress is one of the most damaging environmental stresses; it affects the water balance through stomatal conductance [47]. In glycophytes, salinity stress reduces stomatal conductance, leading to a decrease in the photosynthetic and transpiration rates [48]. In halophytes, this reduction is less [49], resulting in their resistance to stress. In our experiments, there seemed to be a clear difference between the tap water control and the 100 mM NaCl treatment (see Figure 6a,b). Figure 6b shows stomatal closure and reduced red fluorescence. This may have been due to the reduced chlorophyll content. In contrast, the samples treated with the microbial biocontrol agents *B. amyloliquefaciens* (B07) (Figure 6c,d) and *P. terrestris* (Y) (Figure 6e,f) showed a similar chlorophyll content under the two experimental conditions with and without salt stress. The stomatal guard cells were visibly turgid, indicating the opening of the stomata [49], as shown in Figure 6d,f. These results indicate that *B. amyloliquefaciens* and *P. terrestris* had a positive effect on the lettuce plants under the salt stress condition.

### 3.2. Effects of Microbial Biocontrol Agents and Biostimulants on Lettuce Roots as Salt Stress Mitigators

The biometric measurements of lettuce root length, branching, and color showed the best values with Activeg (A) as the salt stress mitigant (see Figure 7a and Figure S1). The results obtained showed that in the control (0, tap water only) and in the B07, B07M, and Ra36M treatments, higher values were recorded for lettuce root length and branching. Salt stress mitigation, albeit at different intensities, was observed in response to the Ra36, Y, and A treatments. The lack of a salt stress-mitigating effect was recorded by analyzing both branching (lateral roots) and root surface coloration in the M, S, SM, C, CM, MiC, and MiCM treatments. Only the treatments with microbial biocontrol agents showed root length, branching, and coloration parameters that were in line with those of the tap water control on the 90th day (Figure 7b).

Our results agree with the literature on salt-stressed plant root morphology [50,51,52,53]. Under our experimental conditions, the microbial biocontrol agents B07, Y, and Ra36 (the latter alone or with molasses added) showed a good ability to mitigate the effects of salt stress (NaCl 100 mM) on root system morphology on both the 75th and 90th days of lettuce seedling growth.

### 3.3. Effects of Microbial Biocontrol Agents as Salt Stress Mitigators on Lettuce Soil Microbial Communities

To show whether NaCl stress could alter the microbial biodiversity communities of the lettuce soil pots, we performed an NGS analysis. The NGS results showed that *Proteobacteria* and *Bacteroidota* were among the most representative phyla in the samples under analysis. These were followed by *Firmicutes*, *Acidobacteriota*, and *Actinobacteriota* in lower proportions (Figure 8a). The bacterial phyla mentioned above are widely distributed in different ecosystems. For example, some authors have indicated that *Proteobacteria*, *Bacteroidota*, *Firmicutes*, and *Actinobacteriota* are the dominant phyla in the oral cavity of *Japalura sensu lato* [54]. Other authors have pointed to the sequential involvement of *Bacteroidota* and *Firmicutes* in the degradation of cellulose from rice straw [55]. Furthermore, some researchers have found that in the rumen of sheep treated by gavage with a mixed bacterial suspension of acid-producing strains isolated from cattle rumen [56], the abundance of *Bacteroidota*, *Actinobacteriota*, *Acidobacteriota*, and *Proteobacteria* significantly increased, whereas bacteria of the phylum *Firmicutes* were much less represented. In the examined pot soils, the relative abundance of *Firmicutes* and *Acidobacteriota* decreased with the salt treatment (group 0_NaCl). In the soils that received the strain B07 and NaCl, an increase in *Firmicutes* and a less drastic reduction in *Acidobacteriota* were observed. In general, 54% of *Firmicutes*, 39% of *Proteobacteria*, and 7% of *Actinobacteria* belong to the salt-tolerant PGPBs evaluated in 40 articles [57]. The most dominant genera of halotolerant PGPBs are *Bacillus* and *Pseudomonas*. PGPBs can provide cross-protection against various stresses and increase plant growth through various direct and indirect mechanisms, including by altering the root morphology; obtaining nutrients; synthesizing exopolysaccharides, phytohormones, volatile compounds, and 1-aminocyclopropane-1-carboxylate (ACC) deaminase; altering ion homeostasis; inducing the aggregation of antioxidants and compatible solutes; inducing systemic tolerance; and modulating stress-sensitive genes [57]. Our experiments are consistent with those of authors who included *B. amyloliquefaciens* among the PGPRs [58]. Our experiments showed that the B07 treatment resulted in a reduction in the *Bacteroidota* abundance, whereas the B07 treatment with 100 mM NaCl increased *Actinobacteriota*.

The genus *Flavobacterium* represents a significant fraction of the root- and leaf-associated microbiome in a wide range of plant species [59], and from our experimental data, it appears that *Flavobacterium*, *Dongia*, *Devosia*, and *Bauldia* were among the most represented genera in all of the groups analyzed. In our experiments, the most abundant taxa in the B07 salt treatment samples were the genera *Flavobacterium*, *Devosia*, and *Bauldia*, which are Gram-negative soil bacteria and useful microorganisms in bioremediation [60,61]. For example, treatment with *Flavobacterium crocinum* HYN0056T under drought and salt stress conditions resulted in the enhanced up-regulation of several drought- and salt-inducible genes in *Arabidopsis* [62], and treatment with *Flavobacterium* sp. strain GJW24 improved the resistance of *Arabidopsis* and *Brassica* plants to drought and drought-related salt stress [63]. In general, microorganisms of the genus *Flavobacterium* are also producers of several molecules that can improve plant health [64]. Regarding the genus *Devosia*, there are studies in the literature on the beneficial effect of the flavonoids and microbe-based material (CFS: cell-free supernatant) containing active compounds secreted by *Devosia* sp. SL43, improving the growth of soybean plants under salt stress [65,66]. *Clostridium* reached an average relative abundance of ≈7.98% in soil treated with B07 and salt. For *Clostridium*, Peng et al. [67] observed the stress-induced enrichment of mRNAs encoding osmoprotectants, such as ABC transporters for choline, betaine, glycine betaine, proline betaine, carnitine, and betaine biosynthesis.

The fungal community analysis focused on the two taxonomic levels of the phylum and the genus. *Ascomycota* were the most abundant in the salt-treated samples. In contrast, *Basidiomycota* were relatively abundant in the tap water control samples (Figure 8b). The most abundant genera were *Candida*, *Botrytis*, *Pseudeurotium*, *Plectosphaerella*, *Chrysosporium*, *Cladosporium*, and *Scytalidium* (Figure 9b). The highest concentration of *Candida* was found in the tap water control samples. However, this yeast was still present in the salt-treated samples. This is not surprising, as other authors have studied *Candida* species that only tolerate salt stress [68,69]. In our experiments, the biocontrol agent *P. terrestris* further restricted *Candida*, although it still represented the most abundant genus in the Yeast_NaCl group. In addition to *Candida*, *Botrytis*, *Stemphylium*, *Pseudeurotium*, and *Chrysosporium* were the most abundant genera in the samples treated with *P. terrestris* and salt. Some of the species belonging to the genus *Stemphylium* include microorganisms of a marine origin, which have adapted to grow in salt-rich environments. Other species (e.g., *Stemphylium lycopersici*) have a positive interaction with *Zea mays* roots that allows for an improved yield under saline stress conditions [70]. In both cases, this could explain the surprisingly high relative abundance of the genus under salt stress with the *P. terrestris* treatment as the result of a useful synergic interaction [71]. *Pseuderotium* sp. has also been found on tomato roots as an endophytic and beneficial fungus [72].

The microbiota expression of the total viable microbial community (bacteria, yeasts, fungi, and spore-forming bacteria) is shown in Table 3. The total microbial counts reached higher values in the enrichment treatments (bioaugmentation) with the microbial strains that were selected as biocontrol agents. Therefore, the effects of adding pure yeast cultures induced positive interactions among the microorganisms in the pot soil. The treatment with *P. terrestris* showed an ability to adapt to the biotic and abiotic conditions of the soil over time. This was confirmed by the counts shown in Table 3.

## 4. Materials and Methods

### 4.1. Experimental Design

**Figure 11:**
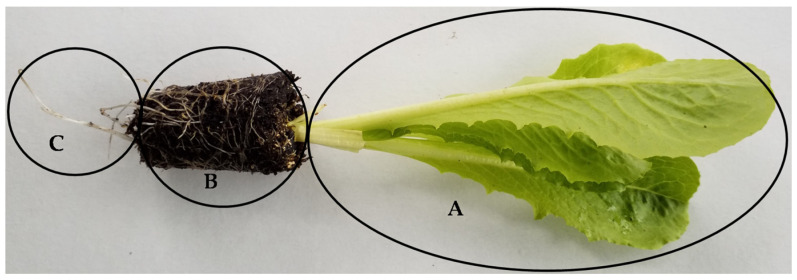
The three different parts of the lettuce plants under investigation: (**A**) leaves, (**B**) peaty substrate, and (**C**) roots.

The trials were carried out using the roots, peaty substrate, and leaves of *L. sativa* L. var *romana* (Figure 11) following the experimental design shown in Figure S2. Lettuce is a species of dicotyledonous angiosperm plant belonging to the family Asteraceae. Its main morphological appearance shows generally thin taproots; basal or root leaves (with the latter forming a basal rosette); and cauline leaves, arranged alternately in the following pattern: the basal ones are short petiolate, and the others are sessile.

The tests were set up according to the following two-factor scheme: (i) the first one was associated with the presence/absence of NaCl, denoted as 0 (tap water, no NaCl) and 1 (NaCl at 100 mM); (ii) the second factor was the type of treatment, and included 14 treatments in addition to the control (tap water), as described in Table 4.

**Figure 12:**
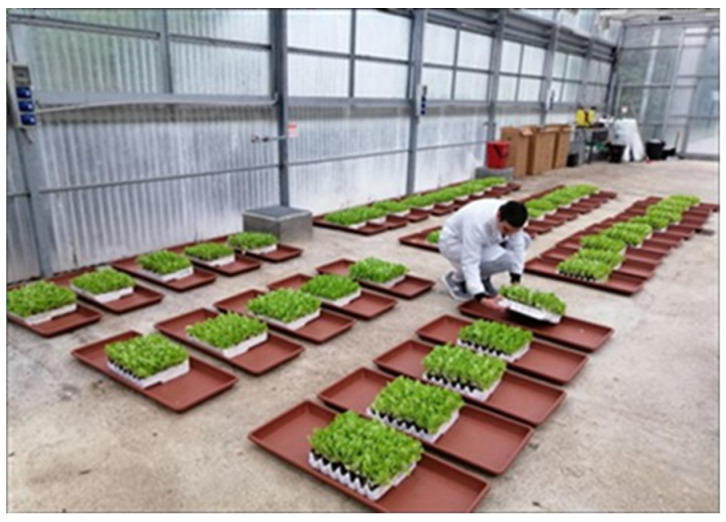
A view of the experimental trial using lettuce growth plots under greenhouse conditions.

The design included about 1400 young lettuce plants in Styrofoam cassettes containing 90 lettuce seedlings. The seedlings were initially grown for 4 weeks post-germination on brown peat substrate enriched with 0.5% of a slow-release fertilizer (NPK: 12-12-12) and furnished by Consorzio Agrario di Isernia, Isernia, Italy. The main physicochemical characteristics of the new soil included a pH of 7.40, an EC of 2.10 mS, available nitrogen in the amount of 18.25 mg kg^−1^, available phosphorus in the amount of 9.20 mg kg^−1^, and available potassium in the amount of 11.50 mg kg^−1^. Each of the thirty treatment blocks was settled with around 45 plants. The experiment was carried out for 4 months in a greenhouse with dimensions of about 30 × 8 × 4 m (long, wide, high), with 240 m^2^ of useful surface; the roof was glass and the lateral walls were transparent methacrylate panels, and there was no heating/cooling control system inside (Figure 12). The ambient conditions during the experimental period were 16–23 °C, with a relative humidity between 70 and 90%; no nutrients were added directly to the substrate. Portable data loggers (RHiLog Escort Data Loggin System Ltd., Auckland, New Zealand) constantly monitored these parameters at the on-site greenhouse at the Dept. Bioscience and Territory, University of Molise, Italy.

The NaCl solution, at a concentration of 100 mM [corresponding to 5.0 Ohm and 1.80 mS m^−1^ of electrical resistivity (ER) and conductivity (EC)], was prepared in a laboratory and obtained by adding 5.80 g L^−1^ (1.30 dS m^−1^) of NaCl to tap water (12.0 Ohm). The defined salt solution can be regarded as stress on the growth of the selected lettuce. The saline solution and biostimulants were administered at regular 7-day intervals, providing a volume of 3.0 L/Styrofoam cassette. The positions of the treatment blocks of the lettuce plants were randomized within the greenhouse, considering their exposure to sunlight, as shown in Figure 12. At the beginning of the experiment and in accordance with the experimental design, 3 L of water was added to each saucer to promote its initial acclimatization in the greenhouse. In this trial, the seedlings were grown under the salt stress produced by 100 mM NaCl. The treatments included microbial suspensions, commercial biostimulants, molasses, compost, and soil extracts that were added at intervals of time to mitigate the effects of saline stress.

### 4.2. Preparation of Biostimulant Solutions and Biocontrol Products

#### 4.2.1. Commercial Biostimulant Solutions

The stock solution of Activeg^®^ (Hydro Fert srl, Bari, Italy) was made with 5.0 g L^−1^ of Activeg in tap water. This biostimulant was obtained through enzymatic hydrolysis from the biomass of *Fabaceae* and was made from high-quality, low-racemization protein hydrolysates containing triacontanol and L-free amino acids, which are active substances ready for use by crops.

The stock solution of Bioalga 15^®^ (Hydro Fert srl, Bari, Italy) was made with 10.0 g L^−1^ of Bioalga 15 in tap water. Bioalga 15 is a growth promoter made up of 15% seaweeds (*Aschophyllum nodosum*), and it also contains natural PGRs (plant growth regulators), enzymes, proteins, vitamins, amino acids, and polysaccharides.

#### 4.2.2. Molasses, Compost, Micro Compost, and Soil Extract Solution

A beet molasses (M) solution, produced by Zuccherificio del Molise, Portocannone, Italy, was prepared as a dilution of 50.0 g L^−1^ in tap water just before use. Its chemical composition was 78.3% dry matter, 13.5% crude protein, and 48% sucrose.

A compost solution was prepared as follows: bio-composted green residues (5.0 kg) (Bio Compost, Compo Group, Munster, Germany) were added to a PVC tank (total volume: 20.0 L) containing 10.0 L of tap water, and the solution was frequently mixed with a rod mixer until 20 min before its use. Then, the supernatant of the compost solution (300 mL) was diluted in 3.0 L of tap water. The diluted solution was used for the treatments.

A micro compost mix (MiC) was created using an enrichment technique, as follows: A total of 100 mL of the compost solution previously created was preliminary subjected to 80 °C for 10 min to induce sporulation within the primitive microbial community. Then, a viable aerobic bacterial count was determined using a 10^−1^ to 10^−8^ serial dilution on plate count agar (PCA, BD Difco, Milan, Italy) that was incubated at 37 °C for 48–72 h. Randomly, five bacterial colonies were selected from the growth on the higher-dilution plates (10^−7^), transferred to fresh PCA media, and stored at 4 °C. When required, the bacterial strains were mixed and inoculated into a flask containing 10 L of sterilized plate count broth and incubated under aerobic conditions at 37 °C overnight. The bacterial growth on the medium was centrifuged at 7000 rpm for 10 min, and the viable bacterial cells were recovered and resuspended in 10 L of tap water before use.

A micro compost mix + M (MiCM) was created by adding a beet molasses solution, as previously reported.

A soil solution was prepared. An agricultural soil sample (Villa Vanda farm, Rosciano, Italy) was collected at a depth of 2–20 cm and sifted with a sterilized sieve (diameter of 2 mm) to remove gravel and plant residues; it was then stored at 4 °C. The soil characteristics were a pH of 7.30, an organic carbon content of 18.40 g kg^−1^, and a Kjeldahl nitrogen content of 0.90 g kg^−1^. The soil (5.0 kg) was added to a PVC tank (total volume of 20.0 L) containing 10.0 L of tap water and frequently mixed with a rod mixer until 20 min before use. Then, the soil supernatant (300 mL) mixed with 3.0 L of tap water was used for the treatments.

#### 4.2.3. Microbial Biocontrol Product Preparation

Biocontrol product solutions based on the B07 and RA36 bacterial strains and the PT22AV yeast strain used in the experiments were prepared according to the references reported in Table 4.

### 4.3. Monitoring Parameters

#### 4.3.1. Mortality Analysis

To evaluate the effectiveness of each treatment in terms of mortality protection, we considered the cumulative incidence risks (CIRs) across the first ten weeks. The cumulative incidence risk at a certain time point is an epidemiological measure, defined as the ratio between the number of deaths within that time point, or the cumulative incidence cases, and the total number of individuals at risk since the beginning of the study, or the population at risk [74]. In epidemiology, the cumulative incidence risk estimates the probability of death within a certain time point.

Concerning our study, for each treatment j (j = 0, 1, …, 14) and for each week t (t = 1, …, 10), we used Y_jt_ to denote the number of plants that died within week t under treatment j and N_j_ to denote the total number of plants under treatment j at the beginning of the experiment. Then, each cumulative incidence risk (CIR) was given by the ratio of the proportion of plants that died within week t under treatment j.

$${\mathrm{CIR}}_{\mathrm{jt}}=\frac{Y_{\mathrm{jt}}}{N_{j}}$$

As a second step, we controlled how significantly different the CIRs of each treatment j were from the CIRs of the control treatment across the ten weeks; that is, for each week t, we proposed the null hypothesis H_0_: CIR_jt_ = CIR_0t_. The statistical test considered was the classical normal Z test, which is generally used to control the difference between two proportions [75]. We considered two levels of significance, 0.01 and 0.001, to accurately differentiate the effectiveness of each treatment.

#### 4.3.2. Biometric Measures

The lettuce plants were tested in non-destructive analyses at intervals of time; in parallel, leaves and then fresh leaf tissues were immediately stored at −80 or −20 °C until use for biochemical analyses. Biometric evaluations, such as the evaluation of a leaf’s growth, were carried out on 13 plants per plot, following an X-shaped design; the biometric measures included the length and width of all of the leaves on each plant, with the mean and standard deviation. This technique guaranteed the continuous growth of the lettuce plants and the respect of our initial experimental design. Therefore, destructive analyses on plants, such as a dry biomass determination or proline assays, were not carried out.

#### 4.3.3. Chlorophyll Content Measurement

**Figure 13:**
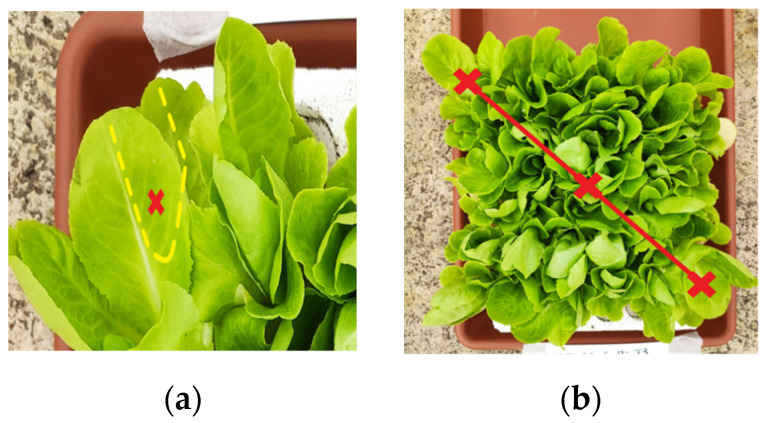
Example of chlorophyll measurement. (**a**) Particular of the leaf portion measured; (**b**) diagonal of the three measures indicated with red crosses.

The relative chlorophyll content was obtained using the AtLeaf CHL PLUS tool (FT Green LLC, Wilmington, DE, USA), a non-destructive indirect method based on the spectral absorption of the leaves [76,77]. The measurements were taken along a diagonal for three points of each seedling block in the middle of the right side of the leaves (Figure 13a,b). The measurements were repeated twice at different times: on the 60th day after the experiment started and from the fourth week after the lettuce plant’s germination (0).

#### 4.3.4. Free Proline Assay

A free proline assay on lettuce leaves was performed using the colorimetric method according to Bates et al. [78,79]. Briefly, a proline standard (60 mg, Sigma-Aldrich, St. Louis, MI, USA), glacial acetic acid, toluene as the extracting solution, and ninhydrin were used. A spectrophotometer was used at 520 nm (UV-1600PC model, VWR^®^, Milan, Italy), with a standard curve ranging from 0.17 to 35 µg/mL.

#### 4.3.5. Morphological and Visual Evaluation of Leaves and Roots

Leaf samples from the lettuce seedlings were collected and fixed on a slide. The autofluorescent images were acquired with a confocal microscope (TCS SP8; Leica, Wetzar, Germany) equipped with 20, 40, and 63× magnification objectives and Leica LAS X software 3.5.5 (Leica Microsystems, Buccinasco, Italy). Visible light (400–600 nm) was used for pigment excitation. A magnification of 20× was employed to visualize the leaf stomata.

The roots were observed at different times; a visual assessment was made by lifting the tray of polystyrene containing the seedlings (see Figure S1). The roots were photographed at each observation time.

Length (1 < 0.5 cm; 2 = 0.6–1.0 cm; 3 = 1.1–2.0 cm; 4 = 2.1–3.0 cm; 5 > 3.1 cm). BRA = branching (a < 0.5 cm; b = 0.6–1.0 cm; c = 1.1–2.0 cm; d = 2.1–3.0 cm; e > 3.1 cm). Color (I, white; II, cream; III, light brown; IV, dark brown; V, black).

Laboratory and portable models for optical microscopy (OM)—Nikon Eclipse E600 model (Nikon Instruments Europe B.V. Amsterdam, The Netherlands)—and stereo microscopy (SM)—Zeiss AxioScope (Carl Zeiss Spa, Milan, Italy)—connected to high-resolution digital cameras were utilized. Samples of leaves and roots obtained at pre-set intervals from the growth trials were examined.

#### 4.3.6. Viable Microbial Count

For the determination of the total viable bacterial count (TVBC), plate count agar (PCA, BD Difco) was used at 37.0 °C for 48–72 h. The spore-forming bacteria (SFB) sample dilutions were preliminary tested at 80 °C for 10 min; they were then inoculated onto Petri dishes with the addition of PCA and incubated at 37 °C for 48–72 h. For the fungal and yeast counts, peptone dextrose agar (PDA, BD Difco) and yeast extract agar (YEA, Biolife Italiana, Milan, Italy) were used at 28 °C for 48 h [80].

#### 4.3.7. Extraction of Genomic DNA from Soil Samples

To assess the bacterial diversity and taxonomic composition through next-generation sequencing (NGS), DNA was extracted at the end of the experimentation from soil samples subjected to the different treatments by using the E.Z.N.A.^®^ Soil DNA Kit (Omega Bio-tek, Norcross, GA, USA) and following the manufacturer’s instructions. For the analysis of fungal communities, DNA was extracted by using the DNeasy 96 PowerSoil Pro QIAcube HT Kit (QIAGEN, Hilden, Germany) according to the manufacturer’s instructions.

#### 4.3.8. 16S rRNA Gene and ITS2 Region Amplicon Library Preparation, Sequencing, and Bioinformatics Analysis

Next-generation sequencing was performed at BMR Genomics srl, Padova, Italy, according to [29]. The sequences generated in the present study were deposited in the NCBI Sequence Read Archive under BioProject PRJNA1140535.

For the fungal communities, the biodiversity within a given sample (alpha diversity) was calculated with the Shannon index and analyzed through the Kruskal–Wallis test. The similarity between samples (beta diversity) was calculated using the Jaccard metric.

A PCoA representation of the beta diversity was performed using QIIME2.

Overall, 18 pot soil samples associated with the lettuce seedlings subjected to different treatments (three replicates for each experimental group) were considered for the study of the micro- and mycobiota (Table S1).

### 4.4. Environmental Impact

To contribute to the reduction in the environmental impacts originating from these experiments, a preliminary assessment of the inputs (energy consumption, reagents, and materials at the lab level) and outputs was initially converted into CO_2_ equivalents (CO_2_ eq.), and then into partial mitigation action through the allocation of the planting of additional plants, according to our previous work [28].

### 4.5. Statistical Analysis

The statistical analysis of the data was conducted in three distinct phases (see Figure S2). The first phase concerned the definition of the experimental design for the collection of data. In this sense, all of the sampling blocks were defined in relation to the treatments (biostimulants or biocontrol agents) and experimental conditions (salt levels), while trying to maintain balance with respect to the number of plants included in each block. All of the results presented in this study are expressed as means ± SD (standard deviation), and an unpaired Student’s *t*-test was used to compare the experimental groups. The statistical analysis was carried out using GraphPad, accessed at https://www.graphpad.com/quickcalcs/ttest2/ (accessed on 23 August 2024) and https://www.socscistatistics.com/tests/studentttest/default2.aspx (accessed on 18 July 2024).

The second phase concerning data analysis was conducted using the free-license computing environment R [81]. The analysis mainly concerned the exploratory aspects with reference to the mortality curve and colorimetric indicators.

The third phase, as shown in Figure S2, represents the final stage, and includes the Environmental Impact, Results, and Discussion Sections.

## 5. Conclusions

The objective of our experiment was to evaluate the capability of selected natural products and microbial biocontrol agents to provide long-term mitigation of salt stress (NaCl 100 mM) in lettuce (*L. sativa* L. var *romana*). This experiment was conducted using Styrofoam cassettes, with each containing 45 lettuce plants. Fourteen different treatments, with and without a salt addition, were analyzed over the long term (90 days) using non-destructive techniques. The results were subjected to a statistical analysis.

The results showed that the biocontrol agents (*P. terrestris*, strain PT22AV; *B. amyloliquefaciens*, strain B07; and *R. aquatilis*, strain 36) and natural compounds, such as some of the biostimulants and molasses, can reduce/mitigate salt stress in *L. sativa* L var. *romana*.

A multifunctional intervention would be able to ensure appropriate responses of the selected biocontrol agents to the following: (i) transplanting stress in the field (efficacy as a biostimulating agent); (ii) environmental stresses, such as climate change, increased salinity, or reduced water availability for crops (efficacy as a bio-attenuating agent); and (iii) antagonistic activity against phytopathogens of horticultural crops (efficacy as a bio-suppressing agent of plant diseases).

Collectively, our data show some aspects of novelty and biological/application interest. In fact, a single biotreatment based on appropriately selected, tested, and effective microorganisms or natural compounds will be used and adopted to ensure growth and plant protection. This will, therefore, be an effective, economically sustainable, and environmentally friendly solution.

## Figures and Tables

**Table 1:** Respective CIRs (in percentage) of treatments and controls.

Test	0	M	S	SM	C	CM	MiC	MiCM	B07	B07M	Ra36	Ra36M	Y	A	B15
H_2_O	31.0	21.4	16.7	14.3	24.5	30.6	34.7	64.3	42.9	16.7	2.4	9.5	19.0	14.3	4.8
NaCl 100 mM	87.8	59.5	90.5	100.0	91.8	36.7	49.0	67.3	38.8	53.1	52.4	31.0	16.7	8.2	62.9

Legend: 0 = control without treatments; M = molasses; S: soil; SM = S + M; C = compost; CM = C + M; MiC = micro compost mix; MiCM = MiC + M; B07 = *B. amyloliquefaciens* strain B07; B07M = B07 + M; Ra36 = *R. aquatilis* strain 36; Ra36M = Ra36 + M; Y = *P. terrestris* strain PT22AV; A = Activeg; B15 = Bioalga 15.

**Table 2:** The biometric data of the whole lettuce plants through the measurement of the leaf size at 60 days. Plants were exposed to NaCl 100 mM, to tap water (control), and to several mitigation treatments. The values are means ± SD (n = 13). Significance was evaluated by Student’s *t*-test. Different letters in the same line indicate statistical differences at *p* < 0.05.

Codes	Biometric Data of Lettuce			
	Height (cm), Mean (±SD)		Width (cm), Mean (±SD)	
	Tap Water	NaCl 100 mM	Tap Water	NaCl 100 mM
0	8.0 (1.1) a	6.7 (0.9) b	3.1 (0.8) a	2.4 (0.4) b
M	8.8 (1.2) a	7.5 (1.0) b	3.6 (1.0) a	3.2 (0.9) a
B07	7.0 (0.8) a	7.1 (0.6) a	2.9 (0.4) a	3.0 (0.4) a
B07M	10.7 (1.4) a	6.9 (0.8) b	3.7 (1.0) a	3.8 (1.1) a
Ra36	12.7 (1.9) a	8.0 (1.1) b	4.3 (1.2) a	3.8 (1.0) a
Ra36M	12.1 (1.7) a	9.0 (1.3) b	3.9 (1.0) a	3.2 (0.7) b
Y	10.0 (1.1) a	11.2 (1.7) b	3.7 (0.6) a	3.7 (0.8) a
A	9.3 (1.2) a	11.7 (1.8) b	3.1 (0.5) a	4.1 (0.4) b

Legend: 0 = control without treatments; M = molasses; B07 = *B. amyloliquefaciens* strain B07; B07M = B07 + M; Ra36 = *R. aquatilis* strain 36; Ra36M = Ra36 + M; Y = *P. terrestris* strain PT22AV; A = Activeg.

**Table 3:** The microbial counts (as log CFU g^−1^ ± SD) analyzed in the treated pot soil exposed to both tap water and salt solution (NaCl 100 mM) on the 90th day. The results are expressed as the mean of three replicates separately analyzed ± SD. The exact value of (*p*) was evaluated by Student’s *t*-test.

Treatments	Microbial Groups	Tap Water Log CFU g^−1^ (±SD)	NaCl 100 mM Log CFU g^−1^ (±SD)	*p*
0	TVBC	5.9 (0.35)	5.0 (0.30)	0.03309
	F and Y	3.4 (0.21)	3.6 (0.26)	0.26481
	SFB	5.2 (0.25)	4.3 (0.30)	0.01960
M	TVBC	5.0 (0.40)	6.4 (0.25)	0.05988
	F and Y	4.7 (0.28)	5.4 (0.40)	0.05635
	SFB	3.6 (0.35)	3.9 (0.36)	0.29379
B07	TVBC	6.7 (0.38)	6.0 (0.46)	0.50818
	F and Y	4.5 (0.43)	4.7 (0.64)	0.60456
	SFB	6.2 (0.47)	5.3 (0.38)	0.07152
B07M	TVBC	6.5 (0.32)	5.6 (0.65)	0.11194
	F and Y	4.4 (0.25)	4.1 (0.24)	0.27961
	SFB	6.0 (0.66)	5.0 (0.40)	0.09997
Ra36	TVBC	7.0 (0.65)	6.7 (0.55)	0.63783
	F and Y	5.5 (0.20)	5.7 (0.30)	0.29601
	SFB	5.2 (0.35)	6.0 (0.40)	0.50334
Ra36M	TVBC	7.2 (0.55)	6.8 (0.40)	0.42308
	F and Y	5.4 (0.22)	5.8 (0.35)	0.13245
	SFB	4.8 (0.21)	6.2 (0.20)	0.00097
Y	TVBC	6.7 (0.65)	6.7 (0.22)	0.90566
	F and Y	6.0 (0.49)	6.9 (0.26)	0.03573
	SFB	6.6 (0.95)	4.5 (0.30)	0.02349
A	TVBC	5.1 (0.35)	6.8 (0.40)	0.00467
	F and Y	4.7 (0.28)	5.4 (0.40)	0.05635
	SFB	3.6 (0.35)	4.9 (0.36)	0.09610

Legend: 0 = control without treatments; M = molasses; B07 = *B. amyloliquefaciens* strain B07; B07M = B07 + M; Ra36 = *R. aquatilis* strain 36; Ra36M = Ra36+ M; Y = *P. terrestris* strain PT22AV; A = Activeg. TVBC = Total Viable Aerobic Bacteria; F and Y = fungi and yeasts; SFB = spore-forming bacteria.

**Table 4:** List of treatments adopted for experimental trials with lettuce plants.

Codes	Treatments	Details
0	Control	No treatments; only tap water or 0.1M salt
M	Molasses	Sugar industry by-product (*Beta vulgaris*)
S	Soil mix	Soil sample extract
SM	Soil mix + M	Soil sample extract + M
C	Compost mix	Commercial compost extract
CM	Compost mix + M	Commercial compost extract + M
MiC	Micro compost mix	Bacterial mixture from compost (C)
MiCM	Micro compost mix + M	Bacterial mixture from compost (C) + M
B07	B07 bacterial strain	Pure culture Dpt. AAA, Unimol, Italy, [73] *B. amyloliquefaciens*
B07M	B07 bacterial strain +M	Pure culture + M
Ra36	Ra36 bacterial strain	Pure culture Dpt. AAA, Unimol, Italy, [27] *R. aquatilis* strain 36
Ra36M	Ra36 bact. strain + M	Pure culture + M
Y	Yeast strain	Pure culture Dpt. AAA, Unimol, Italy, [26] *P. terrestris* PT22AV.
A	Activeg^®^	Commercial biostimulant through enzymatic hydrolysis from *Fabaceae*
B15	Bioalga 15^®^	Commercial biostimulant through extraction from *Ascophillum nodosus*

## Data Availability

Data are contained within the article and Supplementary Materials.

## Supplementary Materials

The following supporting information can be downloaded at: https://www.mdpi.com/article/10.3390/plants13172505/s1; Table S1: Codes assigned to soil samples collected from *L. sativa* L. seedlings for microbial community analyses; Table S2: Number of final reads obtained from NGS analysis for studying taxonomic composition of lettuce soil bacterial communities; Table S3: The number of final reads obtained from the NGS analysis for the study of the taxonomic composition of lettuce soil fungal communities. Figure S1: Example of roots observations of lettuce plants in response to salt stress; Figure S2: Schematic diagram of experimental design.
