# Supplementary material for: Microbial Biocontrol Agents and Natural Products Act as Salt Stress Mitigators in Lactuca sativa L
Source: Plants (Basel). 2024 Sep 6;13(17):2505. doi: 10.3390/plants13172505 (PMC11396915; doi:10.3390/plants13172505)
Supplement: Supplementary file 1 [file plants-13-02505-s001.zip › plants-3158392-supplementary.pdf]

SUPPLEMENTARY MATERIALS

Table S1. Codes assigned to soil samples collected from *L. sativa* L. seedlings for microbial community analyses.

Experimental Group/ Treatment	Sample code
Control group 1<br>seedlings "treated" with water	0_H20_1<br>0_H20_2<br>0_H20_3
Experimental group 1<br>seedlings subject to salt stress - [NaCl] = 100 mM	0_NaCl_1<br>0_NaCl_2<br>0_NaCl_3
Control group 2<br>seedlings "treated" with B07 strain	B07_H20_1<br>B07_H20_2<br>B07_H20_3
Experimental group 2<br>seedlings subject to salt stress "treated" with B07 strain	B07_NaCl_1<br>B07_NaCl_2<br>B07_NaCl_3
Control group 3<br>seedlings "treated" with *P. terrestris* cells	Yeast_H20_1<br>Yeast_H20_2<br>Yeast_H20_3
Experimental group 3<br>seedlings subject to salt stress "treated" with *P. terrestris* cells	Yeast_NaCl_1<br>Yeast_NaCl_2<br>Yeast_NaCl_3

Legend. 0_H2O = Tap water; 0_NaCl = NaCl 100mM solution; B07_H2O = *B. amyloliquefaciens* strain B07 in tap water soil treatment; B07_NaCl = B07 treatment on stressed soil; Yeast_H2O = *P. terrestris* strain PT22AV on tap water control soil; Yeast_NaCl = *P. terrestris* strain PT22AV treatments on stressed soil.

Table S2. Number of final reads obtained from NGS analysis for studying the taxonomic composition of lettuce soil bacterial communities.

Sample code	Number of final reads
0_H2O_1	21549
0_H2O_3	28672
0_NaCl_2	17948
0_NaCl_3	14616
B07_H2O_2	26722
B07_H2O _3	23914
B07_NaCl_1	21818
B07_NaCl_2	29532
B07_NaCl_3	15836

Legend. 0_H2O = Control with tap water; 0_NaCl = Control watered with NaCl 100mM solution; B07_H2O = *B. amyloliquefaciens* strain B07 treatment on soil watered with tap water; B07_NaCl = B07 treatment on stressed soil:

Table S3. Number of final reads obtained from NGS analysis for the study of the taxonomic composition of lettuce soil fungal communities.

Sample code	Number of final reads
0_H2O_1	65776
0_H2O_2	57608
0_H2O_3	55918
0_NaCl_1	48958
0_NaCl_2	70778
0_NaCl_3	52760
B07_H2O_1	52669
B07_H2O_2	38252
B07_H2O_3	45569
B07_NaCl_1	44658
B07_NaCl_2	47750
B07_NaCl_3	43915
Yeast_H2O_1	41938
Yeast_H2O_2	54266
Yeast_H2O_3	54137
Yeast_NaCl_1	51374
Yeast_NaCl_2	74014
Yeast_NaCl_3	62342

Legend. 0_H2O = Control with tap water; 0_NaCl = Control watered with NaCl 100mM solution; B07_H2O = *B. amyloliquefaciens* strain B07 in tap water soil treatment; B07_NaCl = B07 treatment on stressed soil; Yeast_H2O = *P. terrestris* strain PT22AV treatment watered with tap water; Yeast_NaCl = *P. terrestris* strain PT22AV treatments on stressed soil.

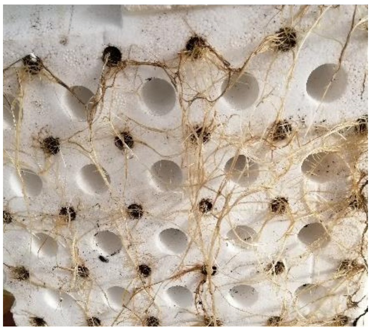

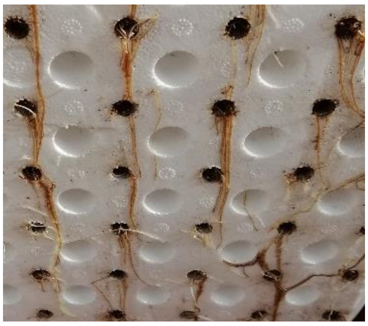

(**a**) (**b**)

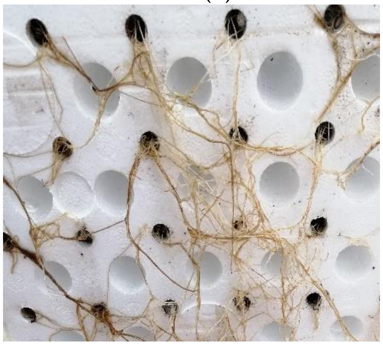

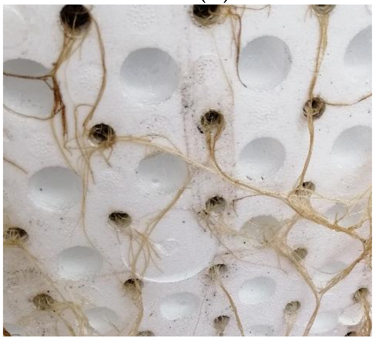

(**c**) (**d**)

Figure S1. Example of roots observations of lettuce plants in response to salt stress (Tap water and 100 mM NaCl), at 75th day. **(a)** Only tap water; **(b)** only salt stress; **(c)** B07 tap water treatment; **(d)** B07 salt stress.

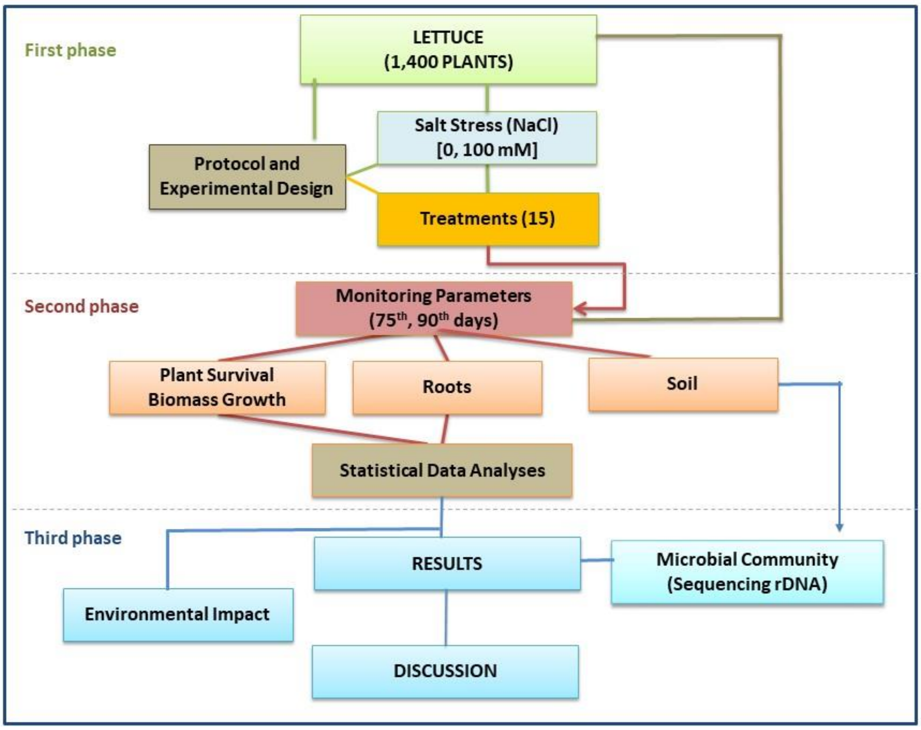

Figure S2. Schematic diagram of experimental design.
